# Efficacy and Safety of Botulinum Toxin vs. Placebo in Depression: A Systematic Review and Meta-Analysis of Randomized Controlled Trials

**DOI:** 10.3389/fpsyt.2020.603087

**Published:** 2020-12-04

**Authors:** Huan Qian, Fangjie Shao, Cameron Lenahan, Anwen Shao, Yingjun Li

**Affiliations:** ^1^Department of Plastic Surgery, The Second Affiliated Hospital, Medical School, Zhejiang University, Hangzhou, China; ^2^Department of Neurosurgery, The Second Affiliated Hospital, Medical School, Zhejiang University, Hangzhou, China; ^3^Burrell College of Osteopathic Medicine, Las Cruces, NM, United States; ^4^School of Public Health, Hangzhou Medical College, Hangzhou, China

**Keywords:** botulinum toxin type A, antidepressant, depression, systematic review, meta-analysis

## Abstract

**Background:** Major depressive disorder (MDD) is a serious mental disorder that represents a substantial public health problem. Several trials have been undertaken to investigate the role of botulinum toxin type A (BTX-A) in the treatment of MDD, but the conclusions were controversial. To examine the efficacy and safety of BTX-A vs. placebo on patients with a clinical diagnosis of MDD, we conducted this systematic review and meta-analysis.

**Methods:** A systematic search was conducted for all relevant randomized controlled trials (RCTs) in PubMed and Web of Science from inception to June 17, 2020. All published studies that investigated the efficacy and safety of BTX-A injections on patients with a clinical diagnosis of MDD were included. The overall effect size was summarized using a random-effects meta-analysis model. The primary outcomes of the present meta-analysis were the changes in depressive rating scale at week 6 after BTX-A injection compared with placebo. The safety of BTX-A injections also was assessed.

**Results:** Five RCTs with a total of 417 participants (189 patients in the BTX-A group, 228 patients in placebo group) were eligible in this meta-analysis. The results indicated an overall positive effect of BTX-A injections for reducing the depressive symptoms of patients with MDD (Hedges' g, −0.82; 95% CI, −1.38 to −0.27) with large effect size. Differences are likely explained by the dose of BTX-As and the gender of the participants. Our findings also highlighted that BTX-A injections were generally well-tolerated, with only mild and temporary adverse events reported.

**Conclusions:** The present meta-analysis provides evidence that BTX-A injections are associated with a statistically significant improvement in depressive symptoms. BTX-A injections are generally safe and may provide a new, alternative option for the treatment of depression.

## Introduction

Major depressive disorder (MDD) is a common and severe mental disorder among the general population. It is related to psychosocial factors, heredity, and changes in the nervous system (1–4). According to the latest data provided by the Global Burden of Disease Study (GBD), ~216 million people suffered from MDD worldwide in 2015 (5). The core symptoms of MDD include sadness, fatigue, and loss of interest or pleasure, which incur a tremendous burden on health and finances (6). Additionally, the high suicide rate associated with severe depression is considered a serious public health concern (7).

Botulinum toxin type A (BTX-A), also known as onabotulinumtoxinA or Botox®, is widely known for its cosmetic efficacy in treating glabellar frown lines (8). It was estimated that more than 1 million cases of BTX-A treatment were reported annually in the United States (9). Emerging evidence suggests that BTX-A injections may exert psychological effects (10, 11). In 2006, a case series first reported the role of BTX-A in the treatment of depression (12). Since this initial report, there has been a growing interest in studying the effect of BTX-A on depression. Wollmer et al. (13) subsequently conducted a randomized double-blind, placebo-controlled trial to explore the effect of BTX-A injections as an adjuvant therapy for MDD. The results showed that depressive symptoms were significantly improved in patients receiving BTX-A injections. The remission and response rates of MDD were also decreased in the BTX-A group compared with the placebo group. Several subsequent trials reported similar results (14, 15). However, a recent large study in 2019 showed that the effects of the high-dosage (50 U) BTX-A injections were similar to effects in the placebo group (16). Given the controversy among different studies, and the growing interest toward complementary and alternative medicine for depression, a systematic review and meta-analysis regarding the efficacy and safety of BTX-A on MDD is worth updating.

Hence, the objective of our study was to comprehensively compile results from the randomized controlled trials (RCTs) and to precisely investigate the efficacy and safety of BTX-A injections as an adjuvant treatment for MDD in comparison to placebo using a meta-analytic methodology. The evidence-based results will benefit further research on MDD.

## Materials and Methods

### Search Strategy

This systematic review and meta-analysis was conducted following the guidelines of the Preferred Reporting Items of Systematic Reviews and Meta-Analyses (PRISMA) statement (17). We systematically searched PubMed and Web of Science to identify all potential literature concerning the role of BTX-A in depression, from inception to June 17, 2020. The following search strategy was adopted: (“*botulinum*” OR “*botox*” OR “*abobotulinumtoxin*” OR “*onabotulinum*” OR “*onabotulinumtoxin*” OR “*botulinumtoxin*” OR “*oculinum*” OR “*dysport*” OR “*botulinotherapy*”) AND (“*antidepressant*” OR “*depression*” OR “*depressive*” OR “*depressed*” OR “*melancholia*” OR “mood disorder^*^” OR “affective disorder^*^” OR “*anxiety*”). The search results were restricted to articles published in English. Moreover, we manually checked the reference citations of all retrieved articles to identify additional publications.

### Inclusion and Exclusion Criteria

Two independent investigators determined potentially relevant studies by screening the titles and abstracts, in duplicate. Next, the papers were assessed to identify eligible studies based on the predefined inclusion criteria. Any discrepancies noted were discussed and resolved with a third investigator.

According to the PICOS criteria, original articles that met the following explicit criteria were eligible: (1) Patients: individuals with the clinical diagnosis of MDD were recruited based on validated and effective diagnostic criteria [e.g., Diagnostic and Statistical Manual of Mental Disorders (DSM-V or DSM-IV)]. We included studies that reported MDD of any severity (mild, moderate, or severe). Studies that recruited patients with depressive symptoms different from MDD or individuals who did not meet the diagnostic thresholds of depression at baseline were excluded; (2) Intervention: BTX-A was administered as an effective intervention for MDD. No restrictions were placed on the form, dosage, or injection site; (3) Comparison: BTX-A injections vs. placebo injections; (4) Outcome: different rating scales of depression were applied to assess the change of depressive symptoms; (5) Study design: only randomized, placebo-controlled trials were included in the present analysis.

### Data Extraction

We extracted effective data from all eligible studies using a standard data extraction checklist. Two independent investigators completed this process. Any discrepancies were discussed and resolved with a third investigator. We extracted the descriptive information, including the first author's name, publication year, country of the participants, interventional duration, study design, severity of depression, diagnosis criteria, dosage of BTX-A, primary outcome measures, and injection region, as well as number, mean age, and gender composition of the participants. Moreover, the pre- and post-treatment means and standard deviations (SDs) of depression scores or the pre- and post-treatment differences of means and SDs of depression scores from each included study were extracted. If a study provided valid data at multiple points after intervention, the time point of the primary outcome was utilized. If any of the eligible studies provided insufficient data, the corresponding authors would be contacted for further information.

### Risk of Bias Assessment

Two investigators used the Cochrane risk of bias tool to assess the methodological quality of each selected study (18). Any discrepancies were discussed and resolved with a third investigator. We evaluated the risk of bias according to the seven following items: random sequence generation, allocation concealment, blinding of participants and personnel, blinding of outcome assessment, incomplete outcome data, selective outcome reporting, and other bias. The potential bias of each item was classified as high, low, or unclear risk. A study was considered high risk of bias if any of the six items were classified as high risk (the item “other bias” was excluded). We assigned an overall low risk of bias if a study was considered low risk in all six items. Otherwise, the study was categorized as overall unclear risk of bias (19).

### Statistical Analysis

To investigate differences in depressive symptoms between BTX-A injections vs. placebo, a meta-analysis method was used to pool extracted data from the included studies. Given the impact of a small sample size on the overall effect size, Hedges' g with corresponding 95% confidence interval (CI) were appropriate to analyze the continuous variables (mean and SDs). When pre-post changes of SDs in depression scores were not reported, an imputed correlation coefficient of 0.5 was used (20), according to the transformation formula in the Cochrane Handbook. When SDs from the original articles were not available, we calculated the estimates from the 95% CI (21). The effect sizes were interpreted under the guidelines (i.e., 0.2, small; 0.5, medium; 0.8 large) (22). We assessed the between-study heterogeneity of effect size using the inconsistency index (*I*^2^) and Cochran *Q*-test (23). *I*^2^ > 50% or *P* < 0.05 was considered statistically significant. The fixed-effects model was applied to calculate the pooled results when no statistically significant heterogeneity was presented; otherwise, a random-effects model was applied to provide more conservative estimates.

Subgroup analysis was conducted using the number of subjects, proportion of females, risk of bias, and measurement tool to investigate the sources of heterogeneity. We performed sensitivity analysis by successive exclusion of each study to test the reliability of the main outcomes. All the statistical analyses of this meta-analysis were performed with STATA software, version 15.1 (Stata Corp, College Station, TX, USA). *P* < 0.05 was considered statistically significant.

## Results

### Search Results and Study Characteristics

**Figure 1:**
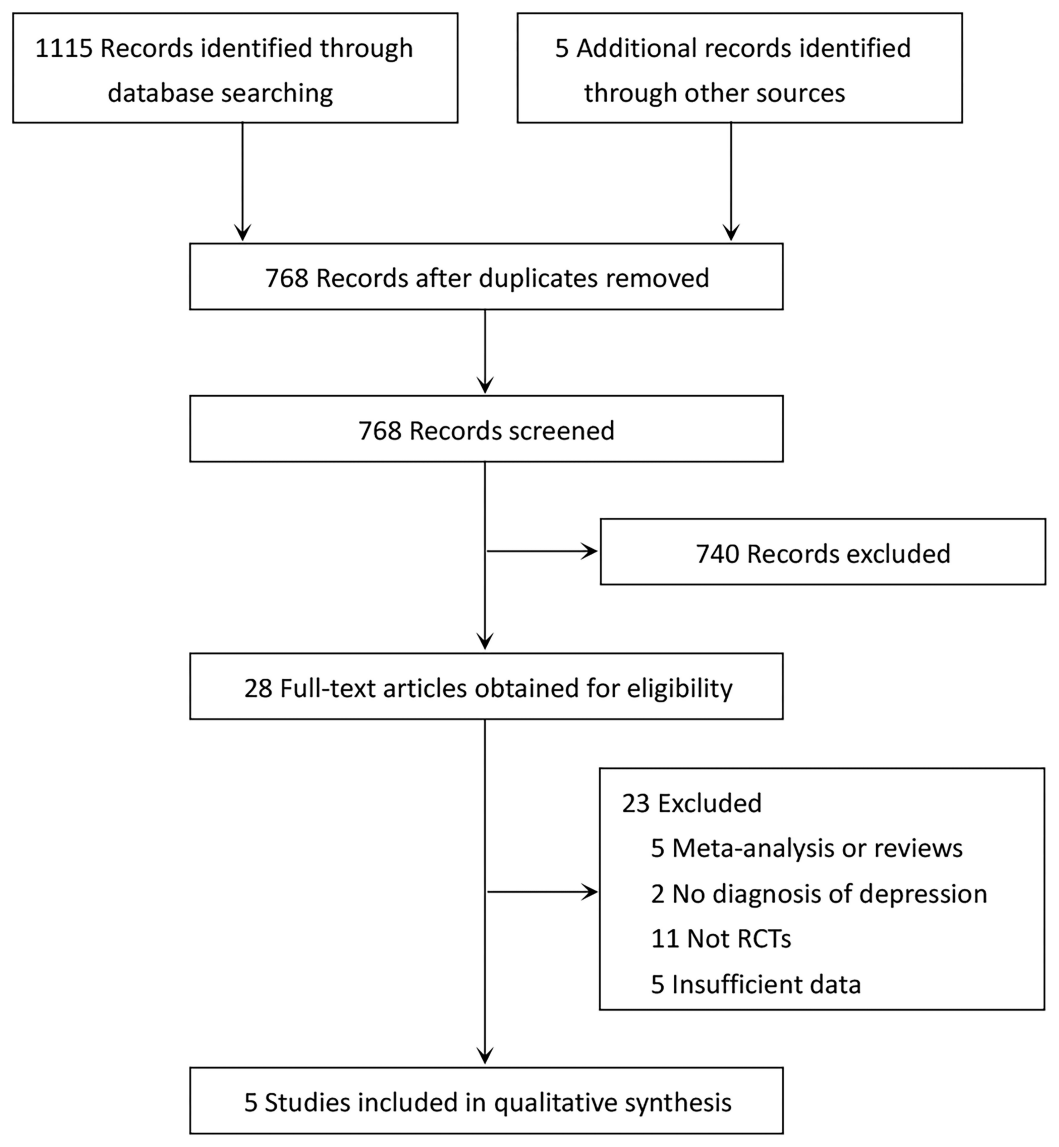
PRISMA diagram of literature search and study selection for systematic review and meta-analysis.

The detailed literature screening process is depicted in Figure 1. Database searching yielded a total of 1,115 related studies, while five potentially eligible studies were obtained from reference citations of retrieved articles. After removing duplicates, 768 studies remained. We excluded 740 completely unrelated articles by evaluating titles and abstracts. For the remaining articles, we obtained the full-text articles for detailed assessment. Twenty-three articles were excluded; the reasons are presented in Figure 1. Finally, five RCTs met the inclusion criteria for this meta-analysis (13–16, 24).

Baseline characteristics of the included studies are listed in Table 1. We identified five eligible articles involving a total of 417 participants (189 patients in the BTX-A group, 228 patients in the placebo group), all of which were randomized, placebo-controlled trials published between 2012 and 2020. Notably, Brin et al. (16) carried out a two-dose parallel groups study of low dosage (30 U) and high dosage (50 U) BTX-A. There were 389 females and 28 males, with a mean (SD) sample size of 70 (44.0) and a mean (SD) age of 46.4 (4.1). The total follow-up period varied from 6 to 24 weeks after a single intervention at baseline. Several countries were involved in the analysis. Three studies were from America, one from Iran, and one from Switzerland and Germany.

**Table 1:** General characteristics of the five RCTs included in the meta-analysis.

**Study ID**	**Country**	**Study Design**	**No. of Subjects (BTX-A, placebo)**	**Mean Age (range)**	**Gender (M/F)**	**Main Diagnosis (diagnostic tool)**	**InterventionDosage (M/F)**	**Duration of Active Treatment (Weeks)**	**Primary Outcome**	**Injection Region**
(16)	USA	Double-blind RCT	Total dosage (30 U): 123 (65, 58)	43.90 (18–65)	Only F	Moderate to severe MDD (DSM-IV)	30 U or 50 U	24	Change in MADRS score at week 6 after injection	Glabellar injections
			Total dosage (50 U): 132 (65, 67)							
(24)	Iran	RCT	28 (14,14)	39.43	14/14	MDD (DSM-V)	NR	6	Change in BDI score at week 6 after injection	NR
(14)	USA	Double-blind RCT	30 (11,19)	49.47 (24–65)	2/28	Mild to severe MDD (DSM-IV)	39 U/29 U	24	Change in HAM-D_21_ score at week 6 after injection	Glabellar injections
(15)	USA	Double-blind RCT	74 (33,41)	48.40 (18–65)	5/69	MDD (DSM-IV)	40 U/29 U	6	Change in MADRS score at week 6 after injection	Glabellar injections
(13)	Switzerland and Germany	Double-blind RCT	30 (15,15)	50.57 (25–65)	7/23	Mild to moderate MMD (DSM-IV)	39 U/29 U	16	Change in HAM-D_17_ score at week 6 after injection	Glabellar injections

*RCT, randomized controlled trial; BTX-A, botulinum toxin type A; MDD, major depressive disorder; M, male; F, female; U, units; DSM, Diagnostic and Statistical Manual of Mental Disorders; MADRS, Montgomery–Åsberg Depression Rating Scale; NR, not reported; BDI, Beck Depression Inventory; HAM-D, Hamilton Depression Rating Scale*.

### Risk of Bias Assessment

A summarization regarding the risk of bias for the five included studies is presented in Table 2. All studies were blinded to participants, investigators, and outcome assessment (24). Only two articles were considered low risk of bias in terms of incomplete outcome data (13, 16), while others were considered high risk of bias because the data regarding the differences in pre- and post-treatment means and SDs were not given directly. For other bias, we only rated one study as high risk of bias (24), but the risk of bias was unclear for four other studies. Three articles were assigned an overall high risk of bias, but the rest were categorized as an overall low or unclear risk of bias.

**Table 2:** Risk of bias assessment of five RCTs included in the meta-analysis.

**Study ID**	**Random Sequence Generation (selection bias)**	**Allocation Concealment (selection bias)**	**Blinding of Participants and Personnel (performance bias)**	**Blinding of Outcome Assessment (detection bias)**	**Incomplete Outcome Data (attrition bias)**	**Selective Reporting (reporting bias)**	**Other Bias**
(16)	Low	Low	Low	Low	Low	Low	Unclear
(15)	Low	Unclear	Low	Low	High	Low	Unclear
(14)	Low	Unclear	Low	Low	High	Low	Unclear
(13)	Low	Low	Low	Low	Low	Low	Unclear
(24)	Unclear	Unclear	High	High	High	Low	High

### Efficacy of BTX-A in MDD

**Figure 2:**
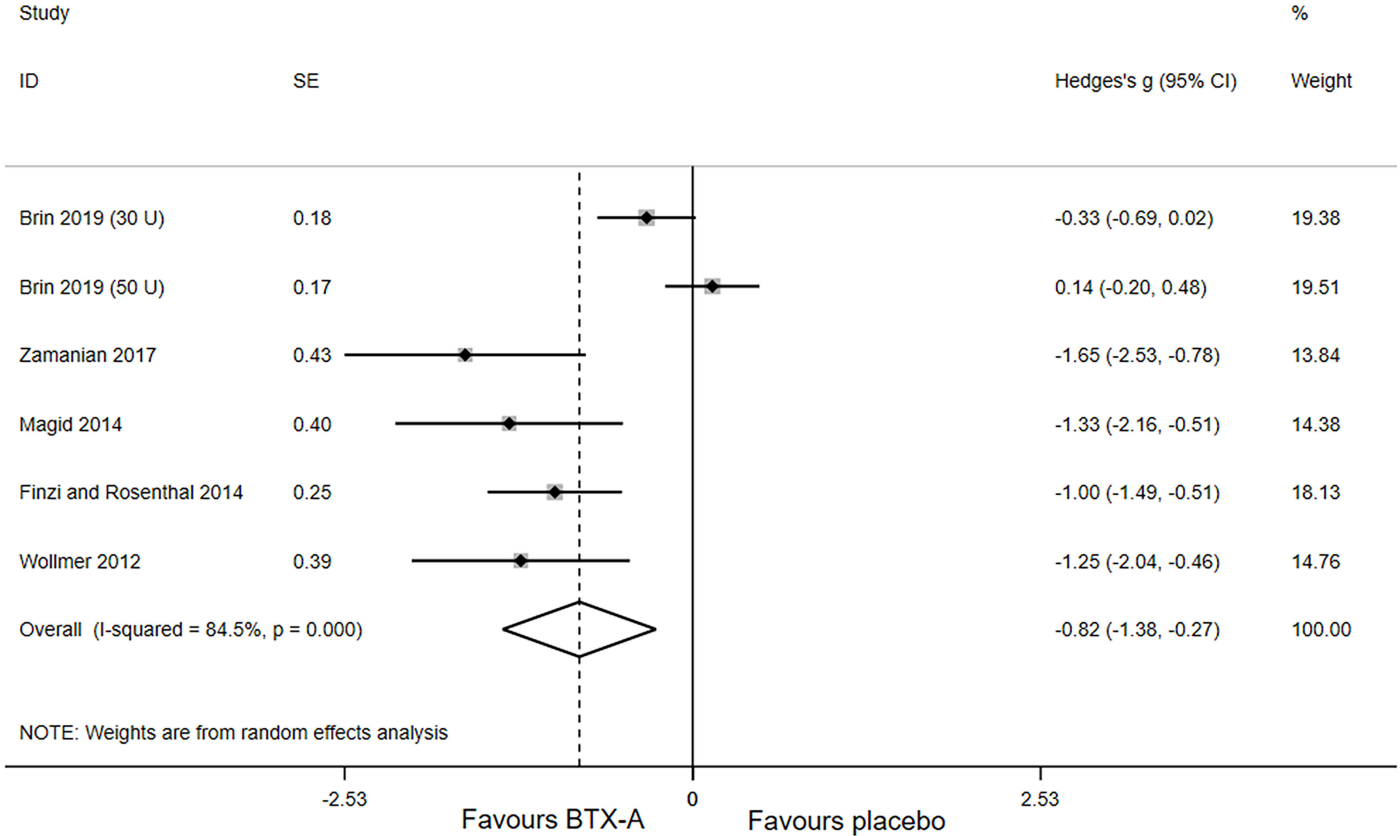
Forest plot of the effect of botulinum toxin type A (BTX-A) injections on reducing the depressive symptoms compared with placebo.

Primary outcomes of all included studies were the changes in depressive rating scale at week 6 after BTX-A injections compared with placebo. The forest plot for the efficacy of BTX-A in MDD is shown in Figure 2. Compared with the placebo group, we found a statistically significant efficacy of BTX-A injections in MDD with a large pooled effect size (Hedge's g, −0.82; 95% CI, −1.38 to −0.27, for the random-effects model). Obvious heterogeneity was observed across the study data (*I*^2^ = 84.5%, *P*-heterogeneity < 0.001).

Subgroup analysis was performed to seek more information. The results stratified by potential modifying factors are shown in Table 3. There was no substantial difference in the overall results of stratified subgroups. After stratifying the number of subjects, the results showed that a relatively large sample size was a momentous source of heterogeneity (*I*^2^ = 86.1%, *P*-heterogeneity = 0.222), but not the small sample size. For studies with a high proportion of females, the pooled Hedge's g was −0.56 (95% CI, −1.15 to 0.03; *I*^2^ = 85.2; *P*-heterogeneity < 0.001), but for studies with a low proportion of females, the pooled Hedges' g was −1.43 (95% CI: −2.02 to −0.84; *I*^2^ = 0.0%; *P*-heterogeneity = 0.503). Moreover, statistically significant heterogeneity was found in studies with low or unclear risk, as well as studies using the Montgomery–Åsberg Depression Rating Scale (MARDS) as the assessment for outcome.

**Table 3:** Subgroup analyses results for the efficacy of BTX-A vs. placebo in MDD.

**Variable**	**Number of Studies**	**Hedges' g (95% CI)**	***P-*value**	***I^**2**^* (%)**	***P-*value^a^**	**Model**
All studies	5	−0.82 (−1.38 to −0.27)	0.004	84.5	< 0.001	Random
Number of subjects						
>52	2	−0.38 (−0.98 to 0.23)	0.222	86.1	0.001	Random
≤ 52	3	−1.40 (−1.87 to −0.92)	<0.001	0.0	0.785	Fixed
Proportion of female						
>90%	3	−0.56 (−1.15 to 0.03)	0.063	85.2	<0.001	Random
≤ 90%	2	−1.43 (−2.02 to −0.84)	<0.001	0.0	0.503	Fixed
Risk of bias						
High risk	3	−1.19 (−1.57 to −0.81)	<0.001	0.0	0.415	Fixed
Low or unclear risk	2	−0.38 (−0.99 to 0.23)	0.117	82.0	0.004	Random
Measurement tool for outcome						
MADRS	2	−0.38 (−0.98 to 0.23)	0.222	86.1	0.001	Random
HAM-D	2	−1.29 (−1.86 to −0.72)	<0.001	0.0	0.885	Fixed
BDI	1	−1.65 (−2.53 to −0.78)	–	–	–	–

*BTX-A, botulinum toxin type A; MDD, major depressive disorder; CI, confidence interval; MADRS, Montgomery–Åsberg Depression Rating Scale; HAM-D, Hamilton Depression Rating Scale; BDI, Beck Depression Inventory*.

a *P-value for heterogeneity within each subgroup*.

We carried out sensitivity analysis to further explore the potential sources of heterogeneity. After excluding one study, (16) the heterogeneity decreased significantly (*I*^2^ = 0.0; *P*-heterogeneity = 0.621), and a more significant effect of BTX-A was observed for the treatment of MDD (Hedges' g, −1.20; 95% CI: −1.54 to −0.86).

### Safety Assessments

In short, the BTX-A injections were well tolerated, and no serious adverse events (AEs) were reported in any of the studies. Magid et al. (14) did not provide data on treatment-related AEs. In the RCT conducted by Zamanian et al. (24) none of the 28 patients with MDD experienced any AEs. The most common AEs, including headache, upper respiratory infection, eyelid ptosis, and injection pain, were noted in the remaining three studies. Brin et al. (16) reported that more than 10% of all patients experienced headaches, but the headaches seemed unrelated to treatment. Moreover, the incidence rates of eyelid ptosis and upper respiratory tract infection added up to 5% in the BTX-A group, which was significantly higher than the placebo group. Transient and mild headaches occurred in 3 of the 74 participants in the study conducted by Finzi and Rosenthal (15), and one patient in the placebo group also complained of nightmares and night terrors. In the study by Wollmer et al. (13), headaches occurred in 40.0 and 26.7% patients in the BTX-A and placebo groups, respectively (Fisher's exact, *P* = 0.700).

## Discussion

This updated meta-analysis identified five independent studies and examined the efficacy and safety of BTX-A injections as an adjuvant treatment in MDD. The findings revealed that BTX-A injections were associated with a significant improvement in depressive symptoms when compared with placebo.

As observed in the sensitivity analysis, the study by Brin et al. (16) was the main source of heterogeneity, which had a significant impact on the summary results. That study used a two-dose parallel design (30 U BTX-A and 50 U BTX-A) and only recruited female patients with a clinical diagnosis of MDD. However, in three other studies [Zamanian et al. (24) did not report the dose of BTX-A injections], 39–40 U and 29 U BTX-A were injected into the glabellar muscles of male and female patients, respectively, and the proportion of male patients varied from 6.8 to 23.3%. Therefore, the dose of BTX-A injections and the gender of the participants may be the main reasons for the difference. In the research conducted by Brin et al. (16), the effect of high-dosage BTX-A injections (50 U) on MDD was similar to the placebo. One possible reason is that more placebo injections may lead to a greater placebo response. In addition, 50 U is higher than the dose commonly used for cosmetic purposes in women. It is possible that the women were actually over-treated and had a worse outcome due to poor cosmetic outcome or some other effect. Due to a limited number of studies, we failed to explore the association between different doses of BTX-A and MDD, as the linear or nonlinear relationship between the dose of BTX-A and MDD was still unknown. Further clinical trials are encouraged to explore the influence that BTX-A dose and gender composition may have in utilizing BTX-A as a treatment for depression.

The results of this meta-analysis showed that BTX-A has a unique advantage in the treatment of MDD. Although the risk of AEs from the BTX-A injections was increased compared to the placebo, the events were mild and brief. The safety of BTX-A was also proven during the treatment of other diseases, such as chronic migraine, primary hyperhidrosis, nocturnal molars, and dystonia (25–28). The long-term effect of a single dose may be conducive to improving compliance and cost-effectiveness. Moreover, the role of BTX-A injections in improving patients' quality of life, self-esteem, and satisfaction was gratifying (29, 30). Thus, BTX-A may provide a new option for the treatment of MDD in the future.

In the present meta-analysis, all trials recruited individuals with a clinical diagnosis of MDD. The effect of BTX-A on depressive symptoms secondary to other diseases or failing to meet the diagnostic criteria of MDD was still unknown. In two RCTs using BTX-A treatment for primary premature ejaculation and chronic tension-type headache, respectively, there was no significant difference in depressive scores between the trial and the control group (31, 32). However, due to the differences in study design and the lack of data, we failed to summarize the results of the two studies. It is worth noting that anxiety disorder is a common comorbidity of depression as nearly 85% of patients with depression are also affected by severe anxiety (33). To date, no RCT has been conducted that has studied the effect of BTX-A on anxiety with/without depression.

Several potential mechanisms have been proposed to explain the beneficial effect of BTX-A in depressive symptoms. The most common theory is the “facial feedback hypothesis” posited by Darwin in 1872, which states that facial expression can affect emotional states (34–36). In 1894, the psychologist James further elaborated this view. He proposed that emotions only change as the body changes, such as blood pressure, heart rate, and of course, expressive behavior (37). The evidence suggests that when corrugator muscles are activated in the forehead, this can lead to negative emotions (38). Furthermore, the study of Schwartz et al. (39) found that the facial muscles of patients with depression are relatively overactive compared to non-depressed individuals. BTX-A injection into the corrugator muscle might block normal sensory feedback from the nerves, especially the left amygdala to the brain (36). Excessive activation of the amygdala was associated with negative emotions (e.g., anger, anxiety, depression, and fear), but the BTX-A reduced the activation of the amygdala by blocking acetylcholine release to the synapses (40), which has a positive effect on mood. In addition, a recently published study has suggested that BTX-A may accomplish antidepressant effects after systemic distribution, although the content of circulating BTX-A is probably very low (41). This theory provides novel insights into the possible mechanism of BTX-A antidepressant effect.

There are some limitations that should be addressed. First, the limited number of studies included was insufficient to support the detection of publication bias. Second, it was difficult to reliably blind participants due to the potential cosmetic effects of BTX-A treatment; therefore, the antidepressant effects of BTX-A may be overestimated. The extent to which the cosmetic effect contributes to the observed improvement of depression symptoms remains unclear. However, a previous work showed that the antidepressant effect of BTX-A lasted for at least 24 weeks, which exceeds the duration of the cosmetic effect on glabellar lines (~12–16 weeks) (14). Third, the diagnosis of MDD to date mostly relies on clinical review using depressive rating scale (e.g., DSM criteria), which may bias the diagnosis results due to the subjectivity. Moreover, there is considerable heterogeneity in the symptoms and severity among different patients even if they were all diagnosed as depression. Fourth, given the statistically significant heterogeneity observed among studies, the use of the random-effects model allowed us to take into consideration the heterogeneity among studies. Finally, the patients included in the study were mainly female, which could be explained by the different interest in cosmetic treatment. Therefore, the results of this study may not be applicable to males. Despite the above limitations, the strengths should also be mentioned. First, considering the impact of the small sample size on the overall effect size, Hedge's g was adopted to analyze the continuous variables. Furthermore, the primary outcome of all the included studies was the changes in depressive rating scale at week 6 after injection, and the injection dose of BTX-A was essentially the same, which improved the comparability. In addition, the main sources of heterogeneity were revealed by sensitivity analysis.

## Conclusion

The findings of this systematic review and meta-analysis confirmed that the glabellar injections of BTX-A were associated with a statistically significant improvement in depressive symptoms. BTX-A injections are generally safe, which may provide a new option for the treatment of MDD. However, further clinical trials are still needed to investigate the antidepressant effect of BTX-A and to explore the underlying mechanisms.

## Data Availability Statement

The raw data supporting the conclusions of this article will be made available by the authors, without undue reservation.

## Author Contributions

YL, AS, and HQ planned and designed the study. YL and HQ conducted the database search and screened studies for inclusion. HQ extracted data. YL, FS, and HQ assessed risk of bias. HQ planned and performed the statistical analysis. HQ wrote the first draft of the manuscript. CL revised the manuscript. All authors contributed to manuscript revision, read, and approved the submitted version.

## Conflict of Interest

The authors declare that the research was conducted in the absence of any commercial or financial relationships that could be construed as a potential conflict of interest.
